# Unraveling the Metabolic and Microbiome Signatures in Fecal Samples of Pregnant Women with Prenatal Depression

**DOI:** 10.3390/metabo15030179

**Published:** 2025-03-06

**Authors:** Jia Li, Peng-Cheng Mei, Na An, Xiao-Xiao Fan, Yan-Qun Liu, Quan-Fei Zhu, Yu-Qi Feng

**Affiliations:** 1 Department of Chemistry, Wuhan University, Wuhan 430072, China; jiajiali@whu.edu.cn (J.L.); pc-mei@whu.edu.cn (P.-C.M.); 2 School of Bioengineering and Health, Wuhan Textile University, Wuhan 430200, China; qf_zhu@whu.edu.cn; 3 Center for Women and Children Health and Metabolism Research, School of Nursing, Wuhan University, 169 Donghu Road, Wuhan 430071, China; 2020203050033@whu.edu.cn (X.-X.F.); liuyanqun@whu.edu.cn (Y.-Q.L.); 4 Frontier Science Center for Immunology and Metabolism, Wuhan University, Wuhan 430071, China

**Keywords:** prenatal depression, gut microbiota, metabolomics, fecal samples, LC-MS analysis

## Abstract

**Background/Objectives**: Prenatal depression (PND) poses a significant threat to the health of both the mother and the developing fetus. Despite its increasing prevalence, the pathophysiology of PND is not yet fully elucidated. **Methods**: In this study, we aimed to investigate the fecal metabolites and gut microbiota in PND patients compared to healthy controls and to explore potential correlations between these factors. **Results**: Through untargeted metabolomics analysis, we identified 75 significantly altered metabolites in PND patients, of which 27 were structurally annotated and implicated key pathways, such as linoleic acid metabolism and phenylalanine, tyrosine, and tryptophan biosynthesis. Notably, two Clostridia-associated enterobacteria, *unclassified_c_Clostridia* and *unclassified_f_Lachnospiraceae*, which were enriched in the PND group, were significantly positively correlated with tyrosine and negatively correlated with multiple sulfated neurosteroids. **Conclusions**: Our findings underscore a robust association between gut microbiota dysbiosis and metabolic disturbances in PND, with specific alterations noted in tyrosine metabolism, sulfated neurosteroid homeostasis, and linoleic acid pathways. These dysregulated metabolites—tyrosine, sulfated neurosteroids, and linoleic acid—may serve as potential diagnostic biomarkers and therapeutic targets. Moreover, their interplay provides new insights into the pathophysiological mechanisms of PND, particularly highlighting the role of gut-brain axis signaling in neuroendocrine dysregulation and inflammatory responses. However, further large-scale studies and animal models are required to validate these findings and explore detailed mechanistic pathways.

## 1. Introduction

Prenatal depression (PND) is an increasingly recognized global public health concern that threatens the physical and mental well-being of pregnant women and potentially has long-term effects on fetal development. Symptoms of PND are diverse, ranging from low mood, fatigue, insomnia, and difficulty concentrating to memory loss, irritability, and cognitive decline [1]. In recent years, the prevalence of PND has shown a rising trend worldwide, with rates ranging from 19% to 25% in resource-constrained settings, compared to 7% to 15% in economically developed regions [2]. This condition not only heightens the risk of pregnancy complications, including preterm birth, miscarriage, and preeclampsia, but also results in impaired fetal development, such as intrauterine growth restriction and suboptimal birth weight trajectories. Additionally, PND can place psychological burdens on the mother and her family, and in severe cases, it elevates the risk of suicide. Moreover, PND may negatively affect the neurocognitive development and behavior of children [3,4,5,6]. Therefore, investigating the contributing factors and pathogenesis of PND is vital for its prevention and treatment, as well as for promoting maternal and child health.

Currently, the exact etiology of PND remains unclear. Some studies suggest that PND is associated with a range of factors, including hormonal fluctuations, dysregulation of the hypothalamic-pituitary-adrenal (HPA) axis, inflammatory responses, abnormal immune system activation, and both genetic and epigenetic factors related to perinatal mood disorders [7,8,9]. Recent studies have begun to explore the role of peripheral organs in the development of depression, with a specific emphasis on the potential link between the gut-brain axis and depression. In this context, the gut microbiota is regarded as a key regulator of this bidirectional communication [10,11,12,13].

The gut microbiota, a complex ecosystem comprising over 10^14^ different microorganisms, has been referred to as the “forgotten organ” of the human body [14,15,16]. Accumulating evidence highlights its crucial role not only in maintaining gastrointestinal health but also in influencing central nervous system function and behavior via the gut-brain axis [17]. Dysbiosis of the gut microbiota may negatively affect brain structure and function, thereby increasing the risk of neuropsychiatric disorders such as schizophrenia, depression, bipolar disorder, and Alzheimer’s disease [13,18,19]. Several studies have highlighted a correlation between depressive states and alterations in specific gut microbial communities, particularly those belonging to the phyla Actinobacteria, Firmicutes, Bacteroidetes, and Proteobacteria [20,21]. For instance, the abundance of Lachnospiraceae, a family within the phylum Firmicutes, was significantly altered in depressed mice and exhibited a strong correlation with inflammatory markers [22], suggesting that the gut microbiome plays a crucial role in linking stress response, inflammation, and the development of depression [23]. The interaction between the host and gut microbiota is mediated by metabolites, underscoring the importance of studying gut microbial metabolites to better understand host-microbiota interactions.

To understand the mechanisms underlying these interactions, metabolomics has emerged as a powerful tool. Metabolomics, a high-throughput analytical approach, enables comprehensive identification and quantification of metabolites, bridging genotypes and phenotypes by capturing genetic, environmental, and developmental influences [24,25,26]. Fecal metabolites, reflecting host-microbiota interactions, offer a non-invasive approach to studying gut-brain axis dynamics [27,28,29,30]. For example, Yuan et al. linked reduced microbial diversity in ulcerative colitis patients with depression/anxiety to dysbiosis and metabolic disturbances [28]. Sinha et al. correlated gut microbiota and metabolites in colorectal cancer, advancing colorectal cancer (CRC) pathogenesis insights [29]. Zhou et al. identified fecal metabolites as biomarkers in obese patients with polycystic ovary syndrome (PCOS), revealing microbiota-metabolite-hormone interactions in hyperandrogenism [30]. These studies highlight fecal metabolite analysis as a powerful tool for understanding neuropsychiatric and metabolic disorders.

Despite these advances, research on the relationship between PND and fecal metabolic phenotypes, as well as gut microbial changes, remains limited. Existing studies have established associations between gut microbial composition—particularly metabolites such as short-chain fatty acids, tryptophan derivatives, and bile acids in PND [31,32,33]. However, these investigations were confined to a narrow range of metabolites, failing to provide a comprehensive overview of the metabolite landscape potentially associated with the disorder. In addition to the metabolites already studied, several other metabolites have shown promise in recent research as being potentially linked to depression [34,35,36,37,38]. For instance, tyrosine, a precursor to neurotransmitters like dopamine, norepinephrine, and epinephrine, plays a crucial role in mood regulation and is closely associated with depression. In individuals with depression, reduced levels of phenylalanine and tyrosine lead to the decreased production of dopamine and other essential neurotransmitters, thereby disrupting mood, cognition, and behavior [34,35,36]. Additionally, disruptions in the steroid sulfate axis, particularly steroid sulfatase (STS) deficiency, may increase the risk of psychiatric disorders during pregnancy [37,38]. Therefore, a comprehensive evaluation of gut microbiota-associated fecal metabolic phenotypes is essential for advancing our understanding of the pathophysiological mechanisms underlying PND.

To address this gap, we conducted an integrated analysis of fecal samples from 77 pregnant women (36 with PND and 41 controls) using untargeted metabolomics via liquid chromatography-mass spectrometry (LC-MS) and 16S rRNA gene sequencing. This multi-omics approach systematically characterizes the gut microbial communities and their associated metabolic profiles in PND patients while exploring their disease associations. By integrating these multi-omics datasets, our study aims to uncover novel insights into the potential mechanisms underlying PND.

## 2. Materials and Methods

### 2.1. Participants

The research adopted a case-control approach, with subject recruitment conducted at the Obstetrics Clinic of Zhongnan Hospital, Wuhan University, between October 2020 and October 2021. Participants were classified into two groups based on their total scores from the Edinburgh Postnatal Depression Scale (EPDS): the PND group (total score ≥ 10) and the control group (total score < 10). A total of 77 pregnant women were included in the study, with 36 in the PND group and 41 in the control group. The specific inclusion and exclusion criteria are detailed in a previously published article [31].

### 2.2. Chemicals and Reagents

Commercial metabolite standards were sourced from three suppliers (Table S1): Aladdin and Meryer (Shanghai, China), along with Sigma-Aldrich (St. Louis, MO, USA). Analytical-grade formic acid was acquired from Sinopharm Chemical Reagent Co., Ltd. (Shanghai, China). LC-MS-grade organic solvents, including methanol (MeOH) and acetonitrile (ACN), were supplied by Merck (Darmstadt, Germany). All aqueous solutions were prepared using ultrapure water generated by a Milli-Q purification system (Millipore, Bedford, MA, USA).

### 2.3. Fecal Sample Collection

Participants were provided with comprehensive instructions on the sampling procedure via detailed explanations, illustrated manuals, and professional video training. Using sterile 30-mL tubes, each subject procured dual biological replicates—designated for microbial community profiling and untargeted metabolomics, respectively. Immediately after collection, samples were placed in insulated containers with ice packs and temporarily stored in a refrigerator at 0 °C. Detailed sample information, including group assignment, identification number, sample type, and collection time, was meticulously recorded. All specimens were then promptly transferred to a −80 °C ultra-low temperature freezer for cryopreservation within 2 h, with ice packs used to maintain low temperatures during transport.

### 2.4. Fecal Sample Preparation

A total of 1 mL of MeOH was added to 20 mg of fecal material. The mixture was homogenized for 3 min in an ice-water bath, followed by sonication for 10 min under the same conditions. Centrifugation at 10,000× *g* (10 min, 4 °C) separated the particulate matter, after which the liquid phase was passed through a 0.22 μm nylon membrane syringe filter (PRECLEANTM, 13 mm diameter) and dried under nitrogen gas. The dried sample was reconstituted in 100 μL of an ACN-water solution (1:1, *v*/*v*) before LC-MS analysis. To ensure methodological reliability, quality control (QC) samples were prepared by pooling 20 μL aliquots from individual fecal extracts and were systematically introduced into the analytical sequence at intervals of every 10 samples throughout the experimental run.

### 2.5. LC-MS Analysis

LC-MS analysis was conducted using a UHPLC-Q-TOF MS system, which included an Agilent 1290 Infinity II liquid chromatography system coupled with an Agilent 6546 Q-TOF mass spectrometer equipped with an Agilent Jet Stream electrospray ionization (ESI, Turbo Ionspray) source (Agilent, Palo Alto, CA, USA). Chromatographic separation was conducted using both reverse-phase liquid chromatography (RPLC) and hydrophilic interaction liquid chromatography (HILIC) modes. For RPLC, a Waters Acquity BEH C18 column (100 × 2.1 mm, 1.7 μm) was used. The flow rate was adjusted to 0.4 mL/min, and the column temperature was maintained at 40 °C. The mobile phases were 0.1% formic acid in water (solvent A) and 0.1% formic acid in ACN (solvent B). The gradient elution program was set for both positive and negative ion modes as follows: 0–1 min, 2% B; 1–23 min, 2–98% B; 23–25 min, 98% B; 25–25.1 min, 98–2% B; 25.1–30 min, 2% B.

For HILIC, a Waters Acquity BEH HILIC column (100 × 2.1 mm, 1.7 μm) was used. The flow rate was adjusted to 0.3 mL/min and the column temperature was maintained at 30 °C. The mobile phases were 0.1% formic acid and 10 mM ammonium formate in water (solvent A) and 0.1% formic acid in ACN (solvent B). The gradient elution program was as follows: 0–2 min, 95% B; 2–18 min, 95–60% B; 18–20 min, 60% B; 20–23 min, 60–95% B; 23–30 min, 95% B. The injection volume was 5 μL.

MS1 data for fecal samples were acquired in full scan mode with a scan rate of 2.5 spectra/s and a scan range of *m/z* 50–1000. The ion source parameters were set as follows: ion transfer tube temperature, 320 °C; fragmentor, 120 V; spray voltage, 3500 V (positive ion mode) and 3000 V (negative ion mode); sheath gas flow rate, 11 L/min; sheath gas temperature, 350 °C; drying gas flow rate, 8 L/min; and nebulizer pressure, 35 psi. MS2 data for differential metabolites were acquired using auto MS/MS mode with an MS scan rate of 3 spectra/s and an MS/MS scan rate of 8 spectra/s. The top six most intense ions per cycle, with an intensity threshold of 10,000, were selected for fragmentation. Collision energies (CE) of 10, 20, 30, and 40 were applied, with a dynamic exclusion time of 0.2 min.

### 2.6. Analysis of Metabolomics Data

Raw data were collected using Agilent MassHunter software (version 10.0). The initial raw data files (.d format) were then converted to abf format with the ABF_Converter tool. Subsequent processing, including peak detection, deconvolution, alignment, blank subtraction, and LOWESS normalization, was carried out using MS-DIAL (version 4.7). Features that failed to meet the 80% rule [39] or exhibited a relative standard deviation exceeding 30% in the QC samples were excluded from the subsequent analysis. Prior to the analysis, the data were log-transformed and auto-scaling to approximate normal distribution.

Differential metabolites were structurally annotated through (a) standard verification, (b) MS^2^ spectral matching using feature-based GNPS molecular networking and SIRIUS 4.9.15 platforms, and (c) MS/MS spectral interpretation. The annotation level followed the guidelines established by the Metabolomics Standards Initiative [40].

### 2.7. 16S rRNA Gene Sequencing

DNA was extracted from fecal samples, followed by PCR amplification, Illumina MiSeq sequencing, and data preprocessing, as outlined in our previous study [31]. The operational taxonomic units (OTUs) were identified from the sequencing data, and their abundance was calculated for both groups of participants. The community composition was analyzed at various taxonomic levels (phylum, genus, etc.), and the relative abundances of microbial taxa at the phylum and genus levels were visualized using bar plots generated on the online platform of Majorbio Cloud Platform.

### 2.8. Statistical Analysis

Descriptive statistics were employed to systematically characterize the demographic and clinical profiles of the study cohort. Intergroup comparisons adopted a dual-mode testing strategy: categorical variables were analyzed using Chi-square tests, while continuous variables underwent Mann-Whitney U tests. Multivariate analyses, including principal component analysis (PCA) and orthogonal partial least squares discriminant analysis (OPLS-DA), were performed using SIMCA 14.1 (Umea, Sweden). Key features contributing to the classification were determined based on the variable importance in projection (VIP) scores. To assess the reliability and performance of the OPLS-DA model, a 200-permutation test was carried out. Metabolic pathway, enrichment, and correlation analyses between metabolites were performed using MetaboAnalyst 5.0. Volcano plots and classification pie charts were generated in Origin, and box plots for metabolites were created using GraphPad Prism 10.3.1. Regression analysis was conducted in R, while correlation networks were visualized with Cytoscape 3.9.1. The graphical abstract was created with BioRender.com.

A linear discriminant analysis effect size (LEfSe) was applied to investigate differences across all classification levels. The relationship between fecal metabolites and gut microbiota was evaluated using R (pheatmap package), with Spearman’s correlation coefficients quantifying the strength of these associations. The results were presented as heatmaps, with color gradients indicating the strength of the correlations between fecal metabolites and gut microbiota.

## 3. Results

### 3.1. Participant Characteristics

A total of 77 pregnant women participated in this study, with 36 PND cases and 41 controls. All participants were married, refrained from alcohol consumption during pregnancy, and carried singleton pregnancies. A significant difference was observed in the proportion of primiparous women between the two groups, with 29.5% in the PND group and 40.3% in the control group (*p* = 0.008). Conversely, no significant differences were found between the groups for factors such as age, height, pregestational BMI, occupation, education, parity, monthly household income, fertility treatment, or schedule (Table 1).

### 3.2. Metabolomics Analysis

**Figure 1:**
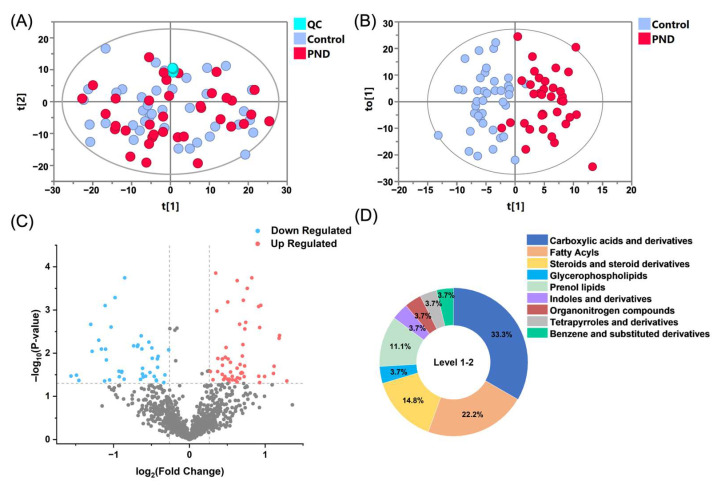
Differential fecal metabolomic signatures between PND and control cohorts. (**A**) PCA and (**B**) OPLS-DA score plots derived from combined RPLC/HILIC-TOF MS analyses in both positive and negative ESI modes. Symbolic representation: PND group (red), controls (blue), and QC samples (cyan). (**C**) A volcano plot illustrates metabolite dysregulation, with up-regulated metabolites shown in red, down-regulated metabolites in blue, and nonsignificant changes in gray. (**D**) Distribution of metabolites across different classes.

An extensive untargeted metabolomics analysis was performed on the fecal samples of the participants, utilizing both RPLC and HILIC coupled with Q-TOF MS in positive and negative ion modes. This approach enabled the delineation of the metabolic differences between the PND group and the control group, facilitating the construction of a comprehensive metabolic landscape. After data processing, 682, 534, 725, and 824 features were detected in the RPLC-ESI (+) TOF-MS, RPLC-ESI (−) TOF-MS, HILIC-ESI (+) TOF-MS, and HILIC-ESI (−) TOF-MS, respectively. To assess analytical reliability, PCA was conducted on the detected metabolites. The resulting score plot demonstrated close aggregation of QC samples (Figure 1A), confirming the robustness and reproducibility of the acquired metabolomic profiles.

### 3.3. Screening and Annotation of Differential Metabolites

To elucidate the metabolic characteristics of pregnant women with PND, we conducted multivariate and univariate statistical analyses on the case and control groups. The OPLS-DA score plots demonstrated a separation between PND patients (red circles) and the controls (blue circles, Figure 1B). The validation plots from the 200-permutation test indicated that the OPLS-DA model had a good fit and predictive ability without overfitting (Figure S1). The volcano plot (Figure 1C) revealed a substantial number of metabolites that were significantly up- or down-regulated in PND patients compared to the controls (false discovery rate (FDR) < 0.05, fold change (FC) > 1.2).

To address the potential influence of the imbalance in the proportion of primiparous women on the results of the metabolite difference analysis, we employed regression analysis to assess the relationship between metabolite abundance and gravidity. In this analysis, gravidity was included as a covariate in the regression model, and all differential metabolites were screened. Based on the results of the regression analysis, metabolites significantly affected by gravidity were excluded. Ultimately, 75 differential metabolites were identified, of which 39 were up-regulated and 36 were down-regulated in PND patients, indicating significant metabolic dysregulation in this PND population.

**Figure 2:**
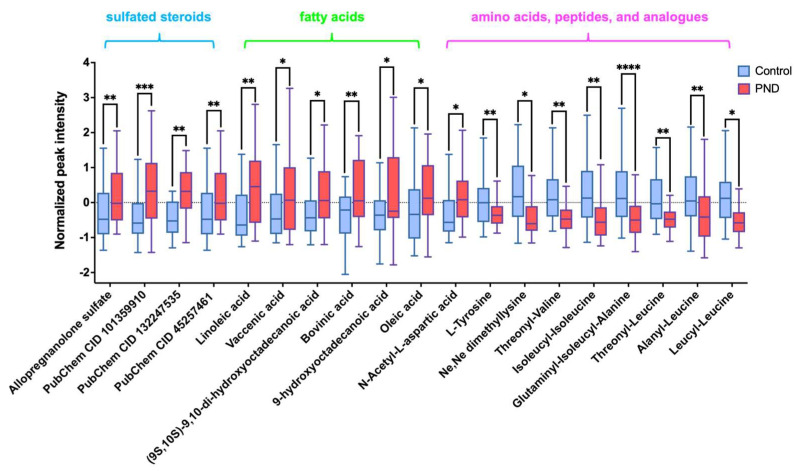
Box plot shows differences between the PND and control groups of four sulfated steroids- Allopregnanolone sulfate, [(3S,8R,9S,10R,13S,14S,17S)-17-acetyl-6-hydroxy-10,13-dimethyl-2,3,4,5,6,7,8,9,11,12,14,15,16,17-tetradecahydro-1H-cyclopenta[a]phenanthren-3-yl] hydrogen sulfate (PubChem CID 101359910), methyl (1S,2S,4bS,7R,8aR)-1,2,4b-trimethyl-7-sulfooxy-1,3,4,4a,5,6,7,8,8a,9,10,10a-dodecahydrophenanthrene-2-carboxylate (PubChem CID 132247535) and [(3R,5S,8R,9S,10S,13S,14S,17R)-17-hydroxy-17-(2-hydroxyacetyl)-10,13-dimethyl-1,2,3,4,5,6,7,8,9,11,12,14,15,16-tetradecahydrocyclopenta[a]phenanthren-3-yl] hydrogen sulfate (PubChem CID 45257461), six fatty acyls-linoleic acid, vaccenic acid, (9S,10S)-9,10-dihydroxyoctadecanoic acid, bovinic acid, 9-hydroxyoctadecanoic acid, and oleic acid), and nine amino acids, peptides, and analogues-L-tyrosine, Ne,Ne dimethyllysine, *N*-acetyl-L-aspartic acid, threonyl-valine, isoleucyl-isoleucine, glutaminyl-isoleucyl-alanine, threonyl-leucine, alanyl-leucine, and leucyl-leucine). *, 0.01 < *p* ≤ 0.05; **, 0.001 < *p* ≤ 0.01; ***, 0.0001< *p* ≤ 0.001, ****, *p* < 0.0001.

Subsequently, a structural annotation of these significantly altered metabolites was performed to explore their potential functions. A total of 27 metabolites were annotated with high confidence (Table S2), of which 6 were confirmed using standards (level 1) and the remaining were matched to MS2 spectra in publicly available databases (level 2). According to the ClassyFire chemical taxonomy, these differential metabolites were primarily classified into nine classes (Figure 1D): carboxylic acids and derivatives (33%), fatty acyls (22%), steroids and steroid derivatives (15%), prenol lipids (11%), glycerophospholipids (4%), and others. Box plots show that the content of fatty acyls, steroids and steroid derivatives (four sulfated steroids), glycerophospholipids, etc., was up-regulated in the PND group compared to the control group (Figure 2 and Figure S2A,B), whereas carboxylic acids and derivatives (nine amino acids, peptides, and analogues), prenol lipids, etc., were down-regulated (Figure 2 and Figure S2C–F).

### 3.4. Correlation Analysis of Differential Metabolites

**Figure 3:**
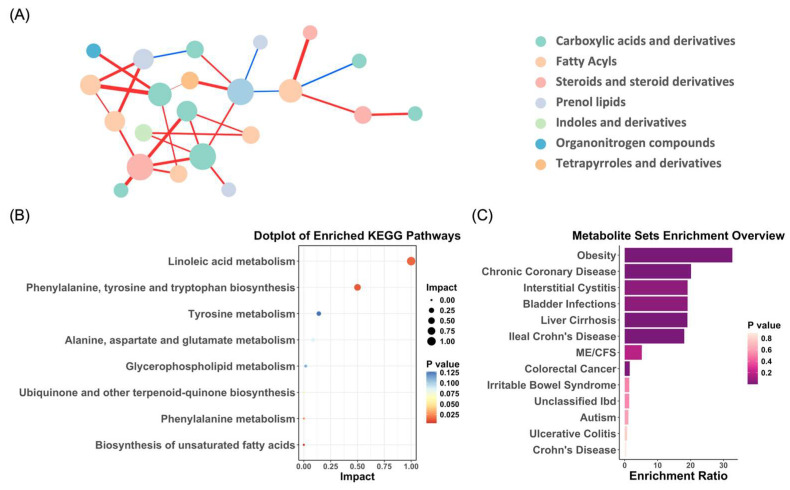
Integrated analysis of differential metabolite network, KEGG pathway enrichment, and disease associations. (**A**) Debiased sparse partial correlation network analysis demonstrating the differential relationships between significantly altered metabolites. Each node represents a metabolite, with its size indicating betweenness centrality, which reflects how often a metabolite appears on the shortest paths between others. The edges represent the strength of partial correlations between metabolites after adjusting for all other variables in the dataset. Red edges indicate positive correlations, while blue edges represent negative correlations. (**B**) KEGG pathway analysis highlighting the significantly altered metabolites in PND. (**C**) Association of PND-related metabolites with human disease states, based on existing metabolomics data.

Using the normalized intensities of the differential metabolites, we explored the interrelationships between significantly varying metabolites using correlation network analysis (Figure 3A). The analysis revealed extensive inter-metabolite and intra-metabolite group interactions, with the carboxylic acids and derivatives showing strong correlations both within and between groups (with the fatty acyls and steroids and steroid derivatives). These findings highlight that even if each compound changes dynamically during gestational depression, there is a highly coordinated metabolite regulatory network.

### 3.5. Pathway and Enrichment Analysis of Metabolites Associated with PND

The Kyoto Encyclopedia of Genes and Genomes (KEGG) pathway and enrichment analysis were conducted on the identified differential metabolites using MetaboAnalyst 5.0. The KEGG pathway analysis revealed that metabolic disruptions associated with PND were predominantly concentrated in pathways, such as linoleic acid metabolism, phenylalanine, tyrosine, and tryptophan biosynthesis, as well as tyrosine metabolism (Figure 3B). Furthermore, the enrichment analysis indicated that several metabolites linked to PND were involved in the pathogenesis of various human diseases, including obesity, chronic coronary disease, interstitial cystitis, and bladder infections (Figure 3C). The observed metabolite changes associated with PND not only impacted the nervous system but also had potential implications for the cardiovascular, immune, urinary, and metabolic systems. This broad spectrum of metabolic alterations suggests that PND may not be confined to a localized pathological process but is closely intertwined with systemic metabolic dysregulation.

### 3.6. Correlation Analysis Between Differential Metabolites and Gut Microbiota

**Figure 4:**
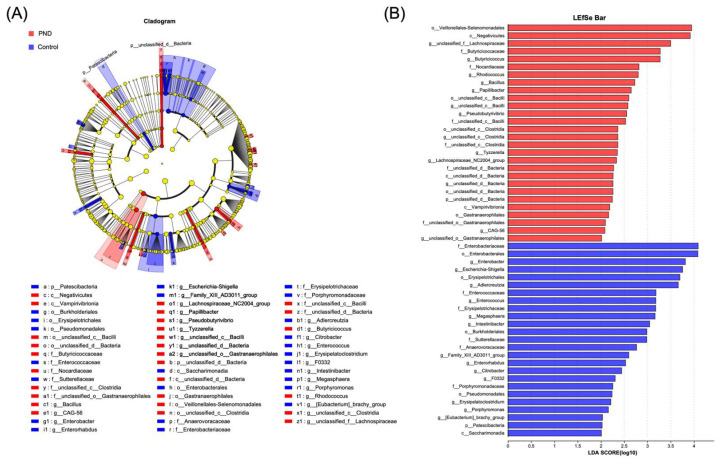
Gut microbiota differential analysis. (**A**) Differences in gut microbiota composition from the phylum to genus levels between the PND and control groups. (**B**) LDA discriminant analysis results. Note: In (**A**), nodes in different colors represent microbial taxa that are significantly enriched in their respective groups and contribute substantially to inter-group differences. Yellow nodes indicate taxa that show no significant difference between groups or have minimal influence on the observed variations. In (**B**), the LDA effect size histogram highlights microbial taxa with significant effects in both groups. The LDA score derived from this analysis indicates that a higher LDA score reflects a greater contribution of species abundance to the observed differences.

The community structure of the intestinal microbiota was analyzed (Figure S3A,B). At the phylum level, Firmicutes, Actinobacteriota, and Bacteroidota were the main components of both groups. At the genus level, in addition to the similarly high abundance of genera (*Blauia*), the PND group showed a significant increase in the genera *Faecalibacterum* and *Subdoigranulum*, and a significant decrease in the genus *Bifidobaterium* compared to the control group. LEfSe analyses were performed to identify key microbiota. Specifically, at the genus level, the PND group exhibited a higher abundance of *unclassified_f_Lachnospiraceae*, *Butyricicoccus*, *Rhodococcus*, *Bacillus*, *Papillibacter*, and *unclassified_c_Bacilli,* whereas the control group exhibited higher abundances of *Enterobacter*, *Escherichia-Shigella*, *Adlercreutzia*, and *Enterococcus* (Figure 4A,B). Collectively, these results suggest that the PND group had a distinct composition of gut microbiota compared to the controls.

**Figure 5:**
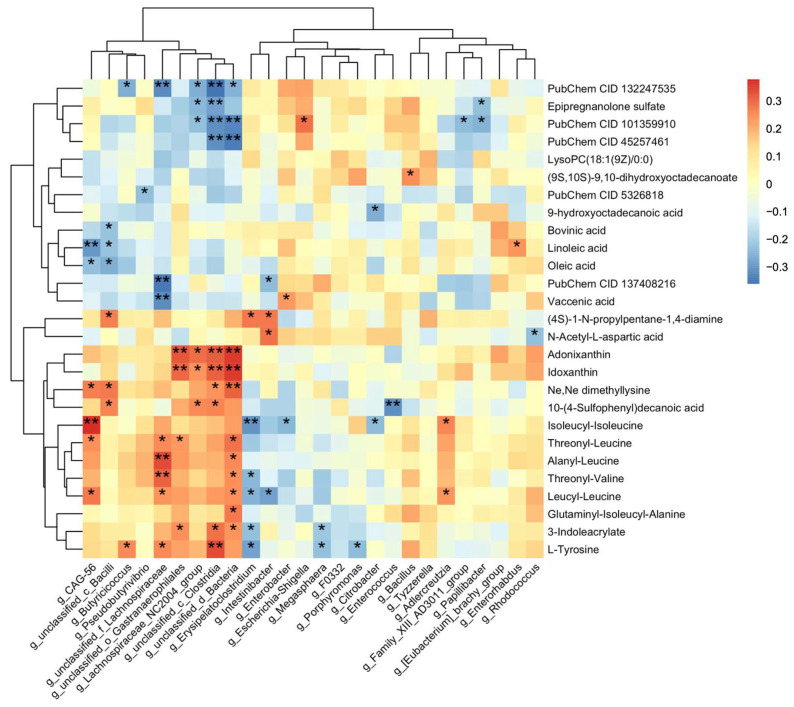
Correlation analysis between differential metabolites and gut microbiota. Heatmap of Spearman’s rank correlation coefficients between the differential metabolites and gut microbiota. PubChem CID 132247535, 101359910, and 45257461 are three sulfated steroids named methyl (1S,2S,4bS,7R,8aR)-1,2,4b-trimethyl-7-sulfooxy-1,3,4,4a,5,6,7,8,8a,9,10,10a-dodecahydrophenanthrene-2-carboxylate, [(3S,8R,9S,10R,13S,14S,17S)-17-acetyl-6-hydroxy-10,13-dimethyl-2,3,4,5,6,7,8,9,11,12,14,15,16,17-tetradecahydro-1H-cyclopenta[a]phenanthren-3-yl] hydrogen sulfate, [(3R,5S,8R,9S,10S,13S,14S,17R)-17-hydroxy-17-(2-hydroxyacetyl)-10,13-dimethyl-1,2,3,4,5,6,7,8,9,11,12,14,15,16-tetradecahydrocyclopenta[a]phenanthren-3-yl] hydrogen sulfate, PubChem 137408216 is a prenol-lipids compound which named (2S)-6-hydroxy-2,5,7-trimethyl-2-[(4R,8R)-4,8,12-trimethyltridecyl]-3H-chromen-4-one. *, 0.01 < *p* < 0.05; **, 0.001 < *p* < 0.01.

To elucidate the potential interactions between gut microbiota and metabolites, a correlation analysis was performed between 27 annotated differential metabolites and 26 genera that exhibited significant differences (Figure 5). The results showed that *unclassified_c_Clostridia*, *unclassified_d_Bacteria*, and *unclassified_f_Lachnospiranceae* were positively correlated with tyrosine and multiple peptides. Additionally, the first two genera were negatively correlated with several sulfated steroids. The unsaturated fatty acid linoleic acid was found to be positively correlated with *CAG_56*. These findings suggest that there may be a potential regulatory relationship between gut microbiota and metabolites.

## 4. Discussion

Neuroactive steroids (NAS) are cholesterol-derived compounds that modulate mood, behavior, and cognition through interactions with neurotransmitter receptors, such as γ-aminobutyric acid (GABA), N-methyl-D-aspartate (NMDA), serotonin, voltage-gated calcium channels, and voltage-dependent anion channels [41,42,43,44]. A significant association has been established between NAS and several neurological disorders, including depression, neuroinflammation, multiple sclerosis, experimental autoimmune encephalomyelitis, epilepsy, and schizophrenia [41,45,46,47]. Particularly during pregnancy, the dysregulation of NAS metabolism is implicated in maternal mental health disorders, with some women exhibiting hormonally sensitive depressive responses to acute increases in NAS levels [48,49,50]. Steroid sulfation and desulfation are critical processes regulating steroid bioactivity, metabolism, and transport, with their balance essential for maintaining the equilibrium between free and conjugated steroids [51,52,53]. In this study, we observed elevated levels of four sulfated neurosteroids in PND patients compared to the controls (Figure 2). This suggests that disruptions in sulfation may alter the bioavailability and activity of NAS, potentially influencing the pathogenesis and progression of PND. Previous research has demonstrated that sulfated pregnanolone inhibits GABAergic neurotransmission in the brain [54]. Furthermore, sulfated progesterone (PMS) and other sulfated hormones may disrupt maternal bile acid (BA) homeostasis during pregnancy [55], potentially leading to cholestasis and symptoms such as insomnia, irritability, and depression [56]. Collectively, these findings underscore the dynamic interplay between sulfated neuroactive steroids and PND, highlighting potential therapeutic targets for future investigations.

The present study also revealed severe disturbances in the linoleic acid metabolism and the aromatic amino acid anabolic pathway in PND patients. Linoleic acid, known as a kind of n-6 polyunsaturated fatty acid, was found at higher levels in PND patients compared to the controls, suggesting a heightened inflammatory response in PND (Figure 2). These findings align with growing evidence linking inflammation to depression pathophysiology [57,58,59], offering insights for future research on pregnancy-related depression mechanisms.

According to the stacked bar plot (Figure S3), the dominant bacterial phyla in the human gastrointestinal (GI) tract were Firmicutes, Bacteroidetes, Proteobacteria, and Actinobacteria. Among them, Firmicutes was the most abundant phylum in both groups, with a significantly higher abundance in the PND patients compared to the controls. Clostridia, one of the most abundant classes within Firmicutes, plays a pivotal role in maintaining gut homeostasis. In recent years, an increasing number of studies have explored its influence on the central nervous system (CNS) through the gut-brain axis [60,61,62,63,64]. In our analysis, the abundance of *unclassified_c_Clostridia* and *unclassified_f_Lachnospiraceae*, both belonging to the class Clostridia, showed a positive correlation with fecal levels of tyrosine and certain peptides (Figure 5). This suggests that these gut microbiotas may play a role in regulating protein metabolism, specifically through the modulation of tyrosine and peptide levels.

Tyrosine is a critical amino acid that serves not only as a building block for protein synthesis but also as a precursor for several neurotransmitters and hormones, including dopamine, adrenaline, and thyroid hormones. These molecules play essential roles in regulating mood, cognitive functions, and stress responses [65,66,67]. In patients with PND, tyrosine levels were significantly lower compared to the controls (Figure 2). Long-term depression rat models have demonstrated that reduced fecal tyrosine levels correlate with decreased tyrosine in the prefrontal cortex, potentially impairing dopamine function and contributing to PND [68,69,70,71,72]. We hypothesize that this reduction may contribute to PND development via the gut-brain axis. Previous studies have shown that the abundance of Lachnospiraceae is positively correlated with tyrosine levels in the central nervous system (CNS) [73], suggesting that Lachnospiraceae may play a role in regulating tyrosine metabolism. Specifically, Lachnospiraceae ferments aromatic amino acids such as tryptophan, tyrosine, and phenylalanine into metabolic products like phenolic acids and indole acids [74]. This fermentation process may alter amino acid metabolic pathways in the gut, leading to decreased tyrosine levels and subsequently impairing dopamine production and release in the CNS. Dysregulation of dopamine signaling can disrupt limbic system function, resulting in hyperexcitability of neurons in anxiety-related brain regions, such as the amygdala and prefrontal cortex. This neuronal hyperactivity may contribute to emotional instability and the development of anxiety disorders [75].

Furthermore, research has demonstrated that the dopamine system significantly influences whole-body energy metabolism by modulating protein metabolism [76,77,78]. An independent regulatory pathway involving dopamine, aminotransferases, cortisol, and the transcription factor CREB has been proposed to coordinate energy metabolism and neural function [76]. In depression, diminished dopamine signaling disrupts the regulatory control over tyrosine aminotransferase (TAT) by impairing D1 receptor-mediated cAMP activity. This disruption redirects tyrosine utilization from neurotransmitter synthesis toward glucose-generating pathways while simultaneously accelerating amino acid breakdown through increased aspartate/alanine transaminase (AST/ALT) activation. Although initially serving as an adaptive response to energy deficits, this metabolic rewiring progressively depletes dopamine precursors and undermines the biosynthesis of synaptic scaffolding proteins and neurotrophic mediators. Consequently, the erosion of synaptic flexibility and cognitive capacity initiates a self-reinforcing loop characterized by neurotransmitter depletion, compensatory metabolic strain, and neural network deterioration. This interconnected pathophysiology underlies the persistent trajectory of depression and its frequent coexistence with metabolic dysregulation, revealing novel therapeutic opportunities at the neuro-metabolic interface.

Additionally, dopamine dysfunction has been implicated in both depression and obesity. In obesity, impaired dopamine signaling can diminish the brain’s response to food-related rewards, leading to overeating and subsequent weight gain. Similarly, in depression, reduced dopamine levels are associated with decreased interest in and pleasure from daily activities [79,80]. Figure 3C indicates that both disorders share overlapping metabolic mechanisms. These findings suggest that Lachnospiraceae, through its metabolic activity, may play a pivotal role in the gut-brain axis by modulating tyrosine levels and neurotransmitters such as dopamine, thereby influencing mood, cognition, and behavior. The interplay between postnatal depression (PND) and obesity, mediated by shared metabolic mechanisms such as dysregulated dopamine signaling, offers new perspectives for developing treatment strategies for both conditions.

Moreover, we found that the abundance of *unclassified_c_Clostridia* was significantly negatively correlated with four sulfated steroids (Figure 5). Specifically, a reduction in *unclassified_c_Clostridia* in PND patients suggests a potential disruption of its role in lowering sulfated steroid levels. The balance between sulfation and desulfation processes, which strongly influences the activity of steroid hormones, is known to depend on enzyme catalysis [81]. We hypothesize that the decline in *unclassified_c_Clostridia* weakens the inhibition of sulfatase activity, leading to elevated levels of sulfated steroids as a compensatory regulatory mechanism. Modulating Clostridia abundance may help restore neurosteroid equilibrium and could serve as a promising therapeutic strategy for PND in the future.

However, this study has several limitations that warrant acknowledgment. First, the sample size was relatively small, comprising only 77 participants, which underscores the necessity for larger-scale studies to corroborate and extend our findings. Second, we did not conduct animal studies to substantiate the novel insights into the role of gut microbiota in PND pathogenesis, indicating a need for further research in this area. Third, the investigation of the relationship between gut microbiota and metabolites was limited to a preliminary level. Future research should delve deeper into how gut microbiota may influence depressive symptoms in individuals with PND. Additionally, exploring the intricate relationship between these metabolites and gut microbiota could offer valuable insights into the mechanisms underlying PND pathogenesis.

## 5. Conclusions

In conclusion, this study offers valuable insights into the altered fecal metabolites and gut microbiota in patients with PND, revealing significant associations between these factors and potential pathophysiological mechanisms underlying PND. We identified key metabolites, including tyrosine, sulfated neurosteroids, and linoleic acid, which are involved in neurotransmitter synthesis, mood regulation, and inflammation. These metabolites may serve as novel biomarkers and therapeutic targets for PND management. The enrichment of Clostridia-associated bacteria, particularly *unclassified_c_Clostridia* and *unclassified_f_Lachnospiraceae*, in the PND group and their correlations with metabolite levels further underscore the importance of gut microbiota in PND. Although these findings provide a solid foundation for future research, large-scale studies and animal models are essential to validate the identified biomarkers and elucidate the detailed mechanisms involved in PND.

## Figures and Tables

**Table 1:** Participant demographic details (N = 77).

Item	Control Group (N = 41)	Prenatal Depression Group (N = 36)	t/χ^2^	*p*
Occupation			NA	0.273 ^a^
Medical	2 (2.6%)	4 (5.2%)		
Non-medical	34 (44.2%)	24 (31.2%)		
Unemployed	5 (6.5%)	8 (10.4%)		
Educational background			2.921	0.232
College degree or below	11 (14.3%)	6 (7.8%)		
Bachelor’s degree	16 (20.8%)	21 (27.3%)		
Master’s degree or above	14 (18.2%)	9 (11.7%)		
Household_per_monthly_income			NA	0.065 ^a^
<5000 yuan/month	4 (5.2%)	2 (2.6%)		
5000–10,000 yuan/month	24 (31.2%)	13 (16.9%)		
≥10,000 yuan/month	13 (16.9)	21 (27.3%)		
Gravidity			9.597	0.008
1	31 (40.3%)	15 (29.5%)		
2	7 (9.1%)	12 (15.6%)		
≥3	3 (3.9%)	9 (11.7%)		
Parity			1.975	0.159
0	35 (45.5%)	25 (32.5%)		
≥1	6 (7.8%)	11 (14.3%)		
Fertilization_way			NA	0.999 ^a^
Natural conception	37 (48.1%)	33 (42.9%)		
Assisted reproductive technology	4 (5.2%)	3 (3.9%)		
Age, years	29.2 ± 3.23	30.53 ± 3.93	1.521	0.133
Height, cm	162.4 ± 4.48	161.68 ± 5.02	−0.743	0.460
Weight at present, kg	64.27 ± 7.07	63.29 ± 7.87	−0.572	0.568
Weight before pregnancy, kg	52.84 ± 6.16	52.98 ± 7.10	0.086	0.931
Pregestational BMI, kg/m2	19.99 ± 1.92	20.24 ± 2.33	−0.223	0.824

Abbreviations: BMI, body mass index; ^a^ means using Fisher’s exact method. Continuous variables demonstrating normal distribution were expressed as mean ± standard deviation (M ± SD), while nonparametric data were characterized by median and interquartile ranges (M [IQR]).

## Data Availability

The original contributions presented in this study are included in the article and supplementary material. Further inquiries can be directed to the corresponding authors.

## Supplementary Materials

The following supporting information can be downloaded at: https://www.mdpi.com/article/10.3390/metabo15030179/s1, Table S1: Standards information; Table S2: Annotated significantly different metabolites in PND feces; Figure S1: Cross-validation plot with a permutation test repeated 200 times of the OPLS-DA score plot; Figure S2: Boxplots show normalized peak intensities of significantly different metabolites; Figure S3: Composition of gut microbiota of pregnant women in the PND group and control group at (A) phylum level and (B) genus level.
