# Supplementary material for: Unraveling the Metabolic and Microbiome Signatures in Fecal Samples of Pregnant Women with Prenatal Depression
Source: Metabolites. 2025 Mar 6;15(3):179. doi: 10.3390/metabo15030179 (PMC11943767; doi:10.3390/metabo15030179)
Supplement: Supplementary file 1 [file metabolites-15-00179-s001.zip › metabolites-3497561-supplementary.pdf]

Supporting information

for

Unraveling the Metabolic and Microbiome Signatures in Fecal Samples of Pregnant Women with Prenatal Depression

Jia Li[1,†], Peng-Cheng Mei[1,†], Na An[1,2,*], Xiao-Xiao Fan[4], Yan-Qun Liu[4], Quan-Fei Zhu[2], Yu-Qi Feng[1,2,3,*]

[1] *Department of Chemistry, Wuhan University, Wuhan 430072, China.*

[2] *School of Bioengineering and Health, Wuhan Textile University, Wuhan 430200, China.*

[3] *Frontier Science Center for Immunology and Metabolism, Wuhan University, Wuhan 430071, China.*

[4] *Center for Women and Children Health and Metabolism Research, School of Nursing, Wuhan University, 169 Donghu Road, Wuhan, 430071, China.*

[†] *These authors contributed equally*

* *Corresponding authors: Yu-Qi Feng. E-mail: yqfeng@whu.edu.cn; Na An. E-mail: naan_anna@whu.edu.cn*

The supporting information includes following items:

Page S3 Table S1 Standards information

Page S4-6 Table S2 Annotated significantly different metabolites in PND feces

Page S7 Figure S1 Cross-validation plot with a permutation test repeated 200 times of the OPLS-DA score plot.

Page S8 Figure S2 Boxplots show normalized peak intensities of metabolites, including (A) Glycerophospholipids: LysoPC(18:1(9Z)/0:0). (B) Tetrapyrroles and derivatives: 3-[18-(2-carboxyethyl)-8-ethyl-3,7,12,13,17-pentamethyl-21,22-dihydroporphyrin-2-yl]propanoic acid (C) Indoles and derivatives: 3-Indoleacrylate. (D) Organonitrogen compounds: (4S)-N-propylpentane-1,4-diamine. (E) Prenol lipids: Adonixanthin, Idoxanthin, and (2S)-6-hydroxy-2,5,7-trimethyl-2-[(4R,8R)-4,8,12-trimethyltridecyl]-3H-chromen-4-one. (F) Benzene and substituted derivatives: 10-(4-Sulfophenyl)decanoic acid. *, 0.01 < p < 0.05; **, 0.001 < p < 0.01; ***, p < 0.001.

Page S9 Figure S3 Composition of gut microbiota of pregnant women in the PND group and control group at (A) phylum level and (B) genus level.

Table S1 Standards information

Name	CAS number	Formula	Monoisotopic mass
Linoleic acid	60-33-3	$C_{18}H_{32}O_2$	280.2402
Vaccenic acid	693-72-1	$C_{18}H_{34}O_2$	282.2558
N-Acetyl-L-aspartic acid	997-55-7	$C_6H_9NO_5$	175.0480
L-Tyrosine	60-18-4	$C_9H_{11}NO_3$	181.0738
Ne,Ne dimethyllysine	19728-74-6	$C_8H_{18}N_2O_2$	174.1368
(3beta)-Allopregnanolone sulfate sodium salt	215996-42-2	$C_{21}H_{33}NaO_5S$	420.1946
Oleic acid	112-80-1	$C_{18}H_{34}O_2$	282.2558
Threonyl-Leucine	50299-12-2	$C_{10}H_{20}N_2O_4$	232.1423
3-Indoleacrylate	29953-71-7	$C_{11}H_9NO_2$	187.0633

Table S2 Annotated significantly different metabolites in PND feces

Name	*m/z*	RT	Mode	VIP	FC	*p*	Formula	HMDB ID	PubChem CID	Subclass	Class	Level
Linoleic acid	279.2329	19.84	RPLC-ESI(−)	3.0	1.6	1.13E-03	C18H32O2	HMDB0000673	5280450	Lineolic acids and derivatives	Fatty Acyls	1
Vaccenic acid	281.2490	21.30	RPLC-ESI(−)	2.9	1.5	1.63E-02	C18H34O2	HMDB0002080	5281127	Fatty acids and conjugates	Fatty Acyls	1
Oleic acid	281.2485	21.12	RPLC-ESI(−)	2.3	1.2	4.07E-02	C18H34O2	HMDB0000207	445639	Fatty acids and conjugates	Fatty Acyls	2
(9S,10S)-9,10-dihydroxyoctadecanoate	317.2695	14.35	RPLC-ESI(+)	2.1	1.6	1.28E-02	C18H36O4	HMDB0059633	12235230	Fatty acids and conjugates	Fatty Acyls	2
Bovinic acid	281.2483	19.92	RPLC-ESI(+)	2.7	1.3	8.27E-03	C18H32O2	HMDB0003797	5280644	Lineolic acids and derivatives	Fatty Acyls	2
9-hydroxyoctadecanoic acid	323.2562	17.42	RPLC-ESI(+)	2.5	1.3	3.76E-02	C18H36O3	HMDB0061661	9570127	Fatty acids and conjugates	Fatty Acyls	2
N-Acetyl-L-aspartic acid	174.0415	0.75	RPLC-ESI(−)	1.6	1.6	2.32E-02	C6H9NO5	HMDB0000812	65065	Amino acids, peptides, and analogues	Carboxylic acids and derivatives	1
L-Tyrosine	180.0669	7.63	HILIC-ESI(−)	2.0	0.6	2.62E-02	C9H11NO3	HMDB0000158	6057	Amino acids, peptides, and analogues	Carboxylic acids and derivatives	1
Ne,Ne dimethyl-Lysine	175.1450	13.01	HILIC-ESI(+)	2.4	0.5	1.39E-03	C8H18N2O2	HMDB0013287	4478779	Amino acids, peptides, and analogues	Carboxylic acids and derivatives	1
Glutaminyl-Isoleucyl-Alanine	329.1830	8.64	HILIC-ESI(−)	3.1	0.5	5.42E-05	C14H26N4O5		145455096	Amino acids, peptides, and analogues	Carboxylic acids and derivatives	2
Threonyl-Leucine	231.1353	7.54	HILIC-ESI(−)	2.5	0.6	1.74E-03	C10H20N2O4	HMDB0029065	7021828	Amino acids, peptides, and analogues	Carboxylic acids and derivatives	2
Leucyl-Leucine	243.1713	6.48	HILIC-ESI(−)	1.9	0.7	1.08E-02	C12H24N2O3	HMDB0028933	76807	Amino acids, peptides, and analogues	Carboxylic acids and derivatives	2
Alanyl-Leucine	201.1249	7.63	HILIC-ESI(−)	2.3	0.7	5.60E-03	C9H18N2O3	HMDB0028691	96801	Amino acids, peptides, and analogues	Carboxylic acids and derivatives	2
Threonyl-Valine	219.1351	7.89	HILIC-ESI(+)	2.3	0.6	6.70E-03	C9H18N2O4	HMDB0029074	7020902	Amino acids, peptides, and analogues	Carboxylic acids and derivatives	2
Isoleucyl-Isoleucine	245.1867	4.38	RPLC-ESI(+)	2.0	0.7	5.09E-03	C12H24N2O3	HMDB0028932	435718	Amino acids, peptides, and analogues	Carboxylic acids and derivatives	2

Table S2 Annotated significantly different metabolites in PND feces (continued).

Name	*m/z*	RT	Mode	VIP	FC	*p*	Formula	HMDB ID	PubChem CID	Subclass	Class	Level
Allopregnanolone sulfate	397.2050	0.98	HILIC-ESI(−)	1.8	1.5	6.46E-03	C21H34O5S		3080643	Sulfated steroids	Steroids and steroid derivatives	1
[(3R,5S,8R,9S,10S,13S,14S,17R)-17-hydroxy-17-(2-hydroxyacetyl)-10,13-dimethyl-1,2,3,4,5,6,7,8,9,11,12,14,15,16-tetradecahydrocyclopenta[a]phenanthren-3-yl] hydrogen sulfate	429.1951	1.00	HILIC-ESI(−)	2.6	1.4	4.00E-03	C21H34O7S		45257461	Sulfated steroids	Steroids and steroid derivatives	2
methyl (1S,2S,4bS,7R,8aR)-1,2,4b-trimethyl-7-sulfooxy-1,3,4,4a,5,6,7,8,8a,9,10,10a-dodecahydrophenanthrene-2-carboxylate	387.1847	0.98	HILIC-ESI(−)	2.4	1.7	2.67E-03	C19H32O6S		132247535	Sulfated steroids	Steroids and steroid derivatives	2
[(3S,8R,9S,10R,13S,14S,17S)-17-acetyl-6-hydroxy-10,13-dimethyl-2,3,4,5,6,7,8,9,11,12,14,15,16,17-tetradecahydro-1H-cyclopenta[a]phenanthren-3-yl] hydrogen sulfate	413.1999	0.98	HILIC-ESI(−)	3.0	1.8	1.97E-04	C21H34O6S		101359910	Sulfated steroids	Steroids and steroid derivatives	2
Adonixanthin	583.4180	1.01	HILIC-ESI(+)	1.8	0.7	5.39E-03	C40H54O3		16061189	Tetraterpenoids	Prenol lipids	2
Idoxanthin	599.4097	1.04	HILIC-ESI(+)	1.6	0.7	9.74E-03	C40H54O4	HMDB0040656	14056459	Tetraterpenoids	Prenol lipids	2

Table S2 Annotated significantly different metabolites in PND feces (continued).

Name	*m/z*	RT	Mode	VIP	FC	*p*	Formula	HMDB ID	PubChem CID	Subclass	Class	Level
(2S)-6-hydroxy-2,5,7-trimethyl-2-[(4R,8R)-4,8,12-trimethyltridecyl]-3H-chromen-4-one	431.3524	0.98	HILIC-ESI(+)	1.9	1.3	3.86E-02	C28H46O3		137408216	Diterpenoids	Prenol lipids	2
3-Indoleacrylate	188.0716	7.31	HILIC-ESI(+)	2.3	0.6	4.98E-03	C11H9NO2	HMDB0000734	5375048	Indoles	Indoles and derivatives	2
LysoPC(18:1(9Z)/0:0)	522.3592	9.21	HILIC-ESI(+)	2.6	1.6	3.40E-02	C26H52NO7P	HMDB0002815	16081932	Glycerophosphocholines	Glycerophospholipids	2
(4S)-1-N-propylpentane-1,4-diamine	145.1708	12.58	HILIC-ESI(+)	2.1	0.5	4.60E-02	C8H20N2		117677075	Amines	Organonitrogen compounds	2
10-(4-Sulfophenyl)decanoic acid	327.1267	19.00	RPLC-ESI(−)	1.5	0.7	2.97E-02	C16H24O5S		100942782	Benzenesulfonic acids and derivatives	Benzene and substituted derivatives	2
3-[18-(2-carboxyethyl)-8-ethyl-3,7,12,13,17-pentamethyl-21,22-dihydroporphyrin-2-yl]propanoic acid	553.2814	1.07	HILIC-ESI(+)	2.2	1.5	2.87E-02	C33H36N4O4		5326818	Porphyrins	Tetrapyrroles and derivatives	2

m/z, mass-to-charge; RT, retention time; VIP, variable important in the projection; FC, fold change

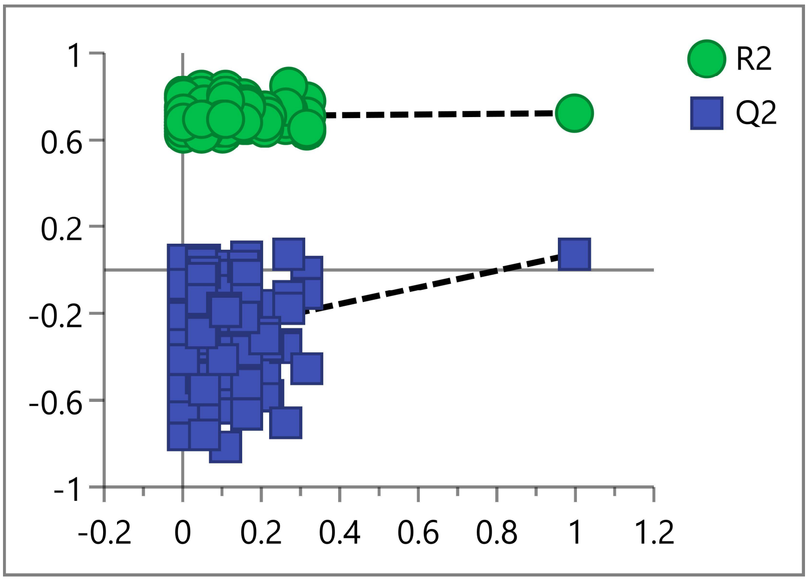

Figure S1 Cross-validation plot with a permutation test repeated 200 times of the OPLS-DA score plot.

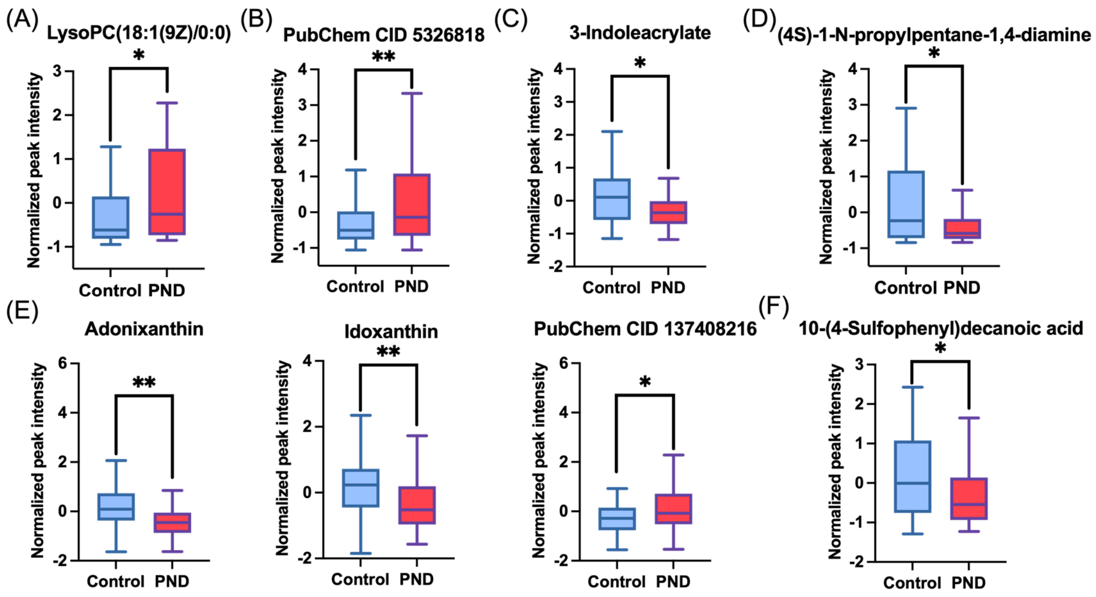

Figure S2 Boxplots show normalized peak intensities of metabolites, including (A) Glycerophospholipids: LysoPC(18:1(9Z)/0:0). (B) Tetrapyrroles and derivatives: 3-[18-(2-carboxyethyl)-8-ethyl-3,7,12,13,17-pentamethyl-21,22-dihydroporphyrin-2-yl]propanoic acid (C) Indoles and derivatives: 3-Indoleacrylate. (D) Organonitrogen compounds: (4S)-N-propylpentane-1,4-diamine. (E) Prenol lipids: Adonixanthin, Idoxanthin, and (2S)-6-hydroxy-2,5,7-trimethyl-2-[(4R,8R)-4,8,12-trimethyltridecyl]-3H-chromen-4-one. (F) Benzene and substituted derivatives: 10-(4-Sulfophenyl)decanoic acid. *, $0.01 < p \leq 0.05$; **, $0.001 < p \leq 0.01$; ***, $0.0001 < p \leq 0.001$, ****, $p < 0.0001$.

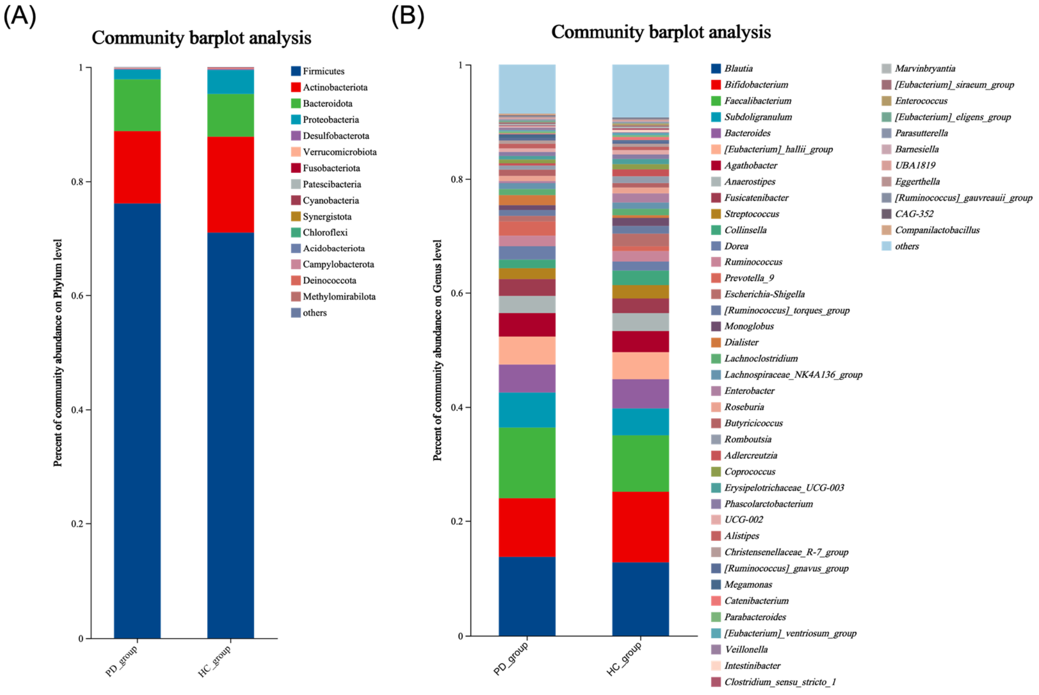

Figure S3 Composition of gut microbiota of pregnant women in the PND group and control group at (A) phylum level and (B) genus level.
